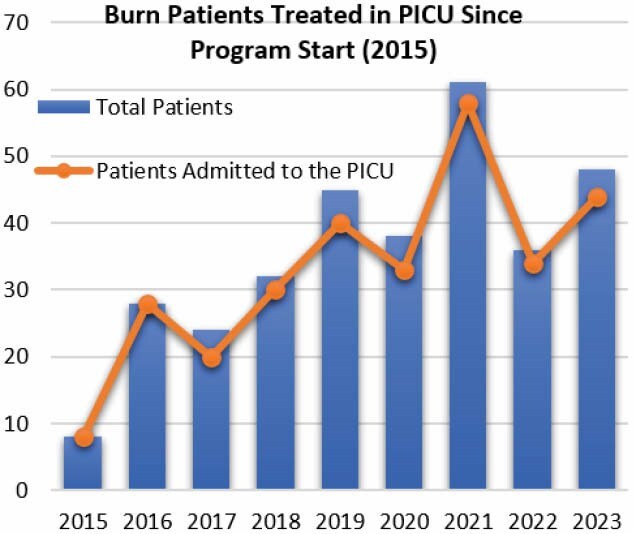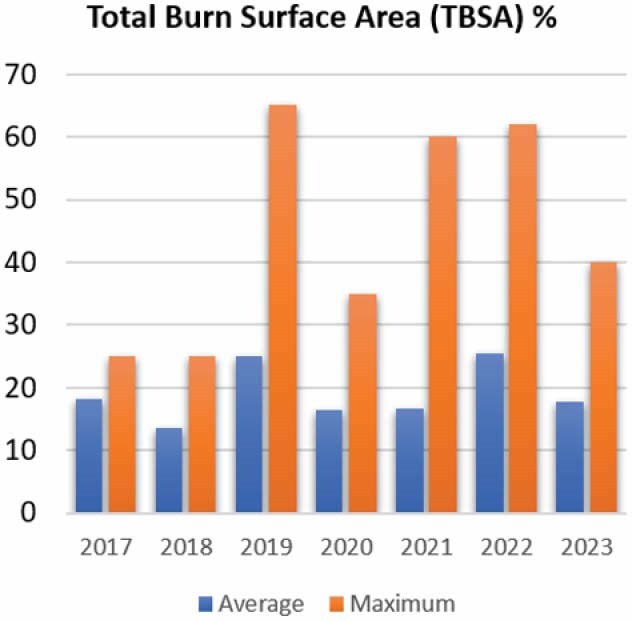# 922 Igniting a Change: Development of a PICU Burn Program

**DOI:** 10.1093/jbcr/iraf019.453

**Published:** 2025-04-01

**Authors:** Christina Carr, Patricia Beck, Kazlauskas Kurt, Moody Ashley

**Affiliations:** University of Florida Health Shands; University of Florida Health Shands; University of Florida Health Shands; University of Florida Health Shands

## Abstract

**Introduction:**

Historically, burn dressing changes for pediatric patients were carried out by burn nurses from the Burn Intensive Care Unit (BICU) daily. Previous research has highlighted the crucial aspects of burn care that matter to pediatric burn patients seeking quality care. To enhance patient care and communication with the families regarding treatment, the decision was made to train nurses in the Pediatric Intensive Care Unit (PICU) to conduct daily burn dressing changes.

**Methods:**

Initially, we identified two experienced nurses in the PICU who were proficient in burn dressing changes. During the initial phase, burn education materials were developed, primarily directed toward the PICU charge nurses. The experienced BICU nurses conducted skills stations for initial training, followed by 24 hours of hands-on experience in the burn unit, where the PICU nurse participated in several burn dressing changes for adult patients. Additionally, the PICU had a part-time nurse who became the designated pediatric champion. This champion shadowed pediatric surgeons, assisted in the outpatient adult and pediatric burn clinics, and spent additional time with the lead nurse in the BICU. The champion burn nurse provided further training to selected primary pediatric burn nurses, contributing to an education process that instilled confidence in the majority of the PICU staff, improving their ability to care for burn victims. Capitalizing on the high turnover during the Covid pandemic, we hired over 67 new nurses and incorporated burn dressing proficiency into the orientation process as an expectation for the PICU nurses.

**Results:**

Currently, we have 70 nurses capable of performing burn dressing changes. These nurses can now educate families on burn care. These nurses can also schedule and coordinate care more effectively.

**Conclusions:**

Furthermore, we observed an increase in the number of burn patients admitted to the PICU, along with an escalation in the severity of burn injuries. Without the transition to having PICU nurses perform burn care, it would have been challenging, if not impossible, for the BICU to manage pediatric burn dressing alongside their primary responsibilities.

**Applicability of Research to Practice:**

Since incorporating the PICU burn program, in-unit burn patients and treatment have quadrupled. Patients from other units are now being pre-scheduled for burn dressings. Burn education continues to improve throughout the unit, putting the PICU in a position to become an asset to high-care burn facilities.

**Funding for the Study:**

N/A